# Contribution of Mitochondrial DNA Variation to Chronic Disease in East Asian Populations

**DOI:** 10.3389/fmolb.2019.00128

**Published:** 2019-11-15

**Authors:** Dayan Sun, Yang Wei, Hong-Xiang Zheng, Li Jin, Jiucun Wang

**Affiliations:** ^1^State Key Laboratory of Genetic Engineering and Collaborative Innovation Center for Genetics and Development, School of Life Sciences, Fudan University, Shanghai, China; ^2^Human Phenome Institute, Fudan University, Shanghai, China; ^3^Ministry of Education Key Laboratory of Contemporary Anthropology, Department of Anthropology and Human Genetics, School of Life Sciences, Fudan University, Shanghai, China

**Keywords:** mitochondrial DNA, variation, haplogroup, chronic disease, East Asian

## Abstract

Mitochondria are the main producers of energy in eukaryotic cells. Mitochondrial dysfunction is associated with specific mitochondrial DNA (mtDNA) variations (haplogroups), and these variations can contribute to human disease. East Asian populations show enrichment of many mitochondrial haplogroups, including A, B, D, G, M7, M8, M9, N9, R9, and exhibit half of the known haplogroups of worldwide. In this review, we summarize the current research in the field of mtDNA variation and associated disease in East Asian populations and discuss the physiological and pathological relevance of mitochondrial biology. mtDNA haplogroups are associated with various metabolic disorders ascribed to altered oxidative phosphorylation. The same mitochondrial haplogroup can show either a negative or positive association with different diseases. Mitochondrial dynamics, mitophagy, and mitochondrial oxidative stress, ultimately influence susceptibility to various diseases. In addition, mitochondrial retrograde signaling pathways may have profound effects on nuclear-mitochondrial interactions, affecting cellular morphology, and function. Other complex networks including proteostasis, mitochondrial unfolded protein response and reactive oxygen species signaling may also play pivotal roles in metabolic performance.

## Introduction

Mitochondria are cytoplasmic organelles of eukaryotic cells that provide more than 90% of the cell's adenosine triphosphate (ATP) through oxidative phosphorylation (OXPHOS) and the mitochondrial electron transport chain (ETC). OXPHOS enzymes include five complexes: complex I (NADH: ubiquinone oxidoreductase), complex II (FADH_2_: succinate dehydrogenase), complex III (coenzyme Q- Cytochrome C [Cyt-C] reductase), complex IV (Cyt-C oxidase), and complex V (F_1_F_0_-ATP synthase). These are embed in the inner mitochondrial membrane. There are about 1,500 proteins that maintain the normal structure and function of mitochondria. Thirteen of these proteins are mtDNA-encoded proteins. The remainder are encoded by nuclear genes and are synthesized in the cytoplasm and transported into mitochondria (Wallace, 2005). Electrons from NADH and FADH2 are transferred to complexes I and II, respectively, and are donated to complex III via ubiquinone. Complex IV then receives electrons from complex III via Cyt-C to reduce molecular oxygen to water. Electrons transferred between these complexes generate a proton gradient across the inner mitochondrial membrane, which is then used by complex V to synthesize ATP from ADP (Wallace, 2013).

mtDNA is continuously synthesized throughout the cell cycle in early human embryonic development. mtDNA has an extremely high mutation rate, presumably due to the lack of histone protection and chronic exposure to mitochondrial reactive oxygen species (ROS). Pathogenic mtDNA mutations include rearrangement mutations (Holt et al., 1988), polypeptide gene missense mutations (Wallace et al., 1988a), and gene mutations (rRNA and tRNA) related to protein synthesis (Wallace et al., 1988b; Shoffner et al., 1990). These mismatches, designated single nucleotide polymorphisms (SNPs) by the Cambridge reference sequence for human mtDNA, determine mitochondrial haplogroups. A mitochondrial haplogroup is a combination of variants that are phylogenetically related (PhyloTree.org-mtDNA tree). A Sub-haplogroup is a sub-branch of haplogroup characterized by new variants, based on the major haplogroup branches. Numerous pathogenic variations in mtDNA or in nuclear DNA (nDNA) encoding mitochondrial proteins may lead to clinically and genetically heterogeneous disorders due to mitochondrial ETC dysfunction (Fang H. et al., 2015; van Rahden et al., 2015; Piekutowska-Abramczuk et al., 2018). Insufficient energy for cardiac and skeletal muscles, brain, liver, and kidney, may lead to metabolic disorders (Lu et al., 2010; Nishigaki et al., 2010; Wang et al., 2010; Liou et al., 2016). Tools for studies of mutant mtDNA have been established by fusing enucleated cells with mitochondria donors, which are called cybrids. Cybrids are useful for studying alterations of mitochondrial function at the cellular level without the influence of the nuclear background (King and Attardi, 1989; Wilkins et al., 2014).

As a multi-ethnic region, East Asia contains nearly half of the known mitochondrial haplogroups, most of which are associated with metabolic and degenerative diseases (Kong et al., 2006; Takasaki, 2008; van Oven and Kayser, 2009). No review has been published discussing the relationship between mtDNA variations and diseases in East Asia. It is important to correlate the East Asian mtDNA variations with different disease to determine their role in pathogenesis. Herein, we review the current research on mtDNA variations and haplogroups in various metabolic diseases and discuss the physiological and pathological relevance of mitochondrial biology in East Asia.

## Mitochondrial Haplogroups Associated With Disease in East Asia

**Figure 1:**
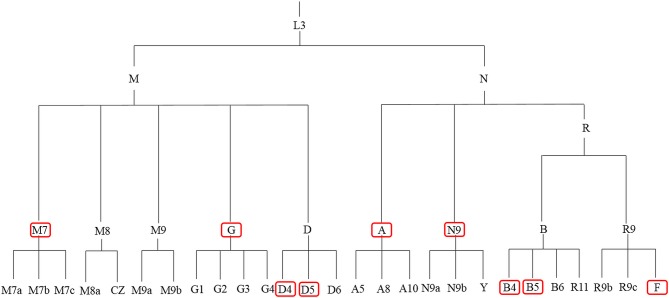
mtDNA tree of the mitochondrial haplogroups in East Asia. The red boxes denote the high frequency mitochondrial haplogroups in East Asia.

Figure 1 and Table 1 summarize previous studies of the East Asia mitochondrial haplogroups and their association with disease Multi-ethnic East Asia populations (Figure 1) account for more than half of the known haplogroups worldwide. Haplogroups M and N are essentially equally represented. Haplogroup M includes sub-haplogroups D4, D5, M7, and G, Haplogroup N includes B4, B5, N9, A, and F. mtDNA variation is not uniform from south to north in East Asia. In the north, more haplogroup M is present, which includes the sub-haplogroups A, C, D4, D5, G, M8, M9, N9, and Z. In the south, more haplogroup N is found, specifically sub-haplogroups B4a,B5a, F, M7, and R9 (Xue et al., 2008). Most of these haplogroups are associated with metabolic and degenerative diseases (Kong et al., 2006; Takasaki, 2008; van Oven and Kayser, 2009). Table 1 shows mitochondrial haplogroups which are closely related to several diseases. Understanding the mechanisms of mitochondrial dysfunction is critical for clinical diagnosis and for development of therapies for patients with mitochondrial diseases.

**Table 1:** Diseases associated with mitochondrial haplogroups in East Asia.

**Haplogroups**		**Associated diseases**	**References**
A		Hearing loss	Wang et al., 2006
		LHON	Ji et al., 2014
		Gastric cancer	Bi et al., 2011
		Oncocytic tumors	Lyu et al., 2019
		Periodontitis	Wang et al., 2015
		Cerebral infarction	Nishigaki et al., 2007a
		Coronary atherosclerosis	Sawabe et al., 2011
		COPD	Zheng et al., 2012
B		Hearing loss	Ying et al., 2015
		Osteoarthritis	Koo et al., 2019
		Acute mountain sickness	Li et al., 2011
	B4	ADHD	Hwang et al., 2018
		Deafness	Lu et al., 2010
	B5	Alzheimer's disease	Bi et al., 2015
		ADHD	Hwang et al., 2018
		Hypertension	Liu et al., 2009
		Leigh syndrome	Hao et al., 2013
C		Deafness	Lu et al., 2010
		OSCC	Lai et al., 2012
	C4a1	Hearing loss	Yuan et al., 2007
		LHON	Zhou et al., 2010
D		Parkinson's Disease	Chen et al., 2015
		Endometrial cancer	Xu et al., 2006
		End-stage renal disease	Zhang et al., 2017
		Lung cancer	Zheng et al., 2012
		Seasonal cold periodontitis	Wang et al., 2015
	D4	Deafness	Liao et al., 2007
		LHON	Qian et al., 2005
		Diabetes mellitus	Liou et al., 2012
		Longevity	Cai et al., 2009
		Nonalcoholic fatty liver disease	Lu et al., 2012
		OSCC	Lai et al., 2012
		Acute myeloid leukemia	Kim et al., 2018
	D5	LHON	Qu et al., 2006
		Diabetes mellitus	Zhong et al., 2014
		Breast cancer	Fang et al., 2010
		Chronic hepatitis B virus infection	Li et al., 2018
		Oncocytic tumors	Lyu et al., 2019
		OSCC	Lai et al., 2012
		Longevity	Alexe et al., 2007
		Metabolic Syndrome	Tanaka et al., 2007
	E2b1	Diabetes mellitus	Loo et al., 2014
F		ADHD	Hwang et al., 2018
		Diabetes mellitus	Niu et al., 2015
		Lung cancer	Fang Y. et al., 2015
		Longevity	Feng et al., 2011
	F1	Hearing loss	Yuan et al., 2007
		LHON	Qian et al., 2005
		Gastric cancer	Bi et al., 2011
		Nasopharyngeal Carcinoma	Hu et al., 2014
	F2	Deafness	Lu et al., 2010
		LHON	Liu et al., 2011
		Gastric cancer	Bi et al., 2011
G		Diabetes mellitus	Liao et al., 2008
		Lung cancer	Zheng et al., 2012
		Osteoarthritis	Fang et al., 2014
	G1	Metabolic Syndrome	Tanaka et al., 2007
H		Diabetes mellitus	Jiang et al., 2017
M		Breast cancer	Fang et al., 2010
		Hepatocellular carcinoma	Zhang et al., 2010
	M1	LHON	Qu et al., 2010
	M7	Lung cancer	Zheng et al., 2012
		Acute mountain sickness	Li et al., 2011
		COPD	Zheng et al., 2012
	M8	Nonalcoholic fatty liver disease	Lu et al., 2012
	M9	Diabetes mellitus	Liao et al., 2008
N	N9a	Hearing loss	Kato et al., 2012
		sCJD	Zhang et al., 2015
		Diabetes mellitus	Fuku et al., 2007
		Metabolic Syndrome	Tanaka et al., 2007
	N9b1	Alzheimer's disease	Takasaki, 2009
R		Hearing loss	Yuan et al., 2007
		Azoospermia	Feng et al., 2013
		Diabetes mellitus	Liao et al., 2008
	R9a	Hearing loss	Young et al., 2005
	R11	LHON	Qu et al., 2010
W	W3a	sCJD	Zhang et al., 2015
Y		LHON	Yu et al., 2010
		Leigh syndrome	Hao et al., 2013
	Y2	Deafness	Ding et al., 2009
		OSCC	Lai et al., 2012
	Z3	LHON	Qu et al., 2010

*mtDNA, mitochondrial DNA; ATP, adenosine triphosphate; OXPHOS, oxidative phosphorylation; ETC, electron transport chain; cyt-C, Cytochrome C; ROS, reactive oxygen species; SNP, single nucleotide polymorphism; nDNA, nuclear DNA; AD, Alzheimer disease; TCA, tricarboxylic acid; PD, Parkinson's disease; ADHD, attention deficit hyperactivity disorder; LHON, Leber's hereditary optic neuropathy; DM, diabetes mellitus; DKD, diabetic kidney disease; HCC, hepatocellular carcinoma; EC, esophageal cancer; HCM, hypertrophic cardiomyopathy; LVNC, left ventricular non-compaction; UPR, unfolded protein response; COPD, chronic obstructive pulmonary disease; sCJD, sporadic Creutzfeldt-Jakob disease; OSCC, oral squamous cell carcinoma*.

### Nervous System Diseases

Neurons need most energy produced from mitochondria to maintain neuron cellular function. In addition, normal mitophagy to eliminate misfolded and aggregated proteins is necessary for neuron cellular function. Mitophagy is a specific process that degrade damaged mitochondria to maintain cellular homeostasis based on the mechanism of autophagy (Youle and Narendra, 2011). Autophagy is dependent on enclosing ubiquitinated proteins in vesicles termed “autophagosomes,” and subsequently lysosomal fusion (Levine and Kroemer, 2008). mtDNA mutation can lead to mitophagy (Dombi et al., 2016) and mitochondrial dysfunction (Dolle et al., 2016; Pera et al., 2017; Ehrnhoefer et al., 2018; Lindqvist et al., 2018; Pereira et al., 2018; Puthumana and Regenold, 2019) have long been reported as pathogenic in psychiatric and neurodegenerative diseases.

#### Alzheimer Disease and Parkinson's Disease

Mitochondrial abnormalities are associated with high levels of amyloid-beta protein in Alzheimer disease (AD) (Wang et al., 2007). Studies found increased tricarboxylic acid (TCA) cycle metabolism in AD patients due to an increased levels of ROS from deficiencies in OXPHOS (Bubber et al., 2005; Carvalho et al., 2009). Other papers suggest that mitochondrial dysfunction may start 3 months before extracellular deposition of amyloid-beta protein, and that progression accelerates with aging (Hauptmann et al., 2009). The mitophagy-related-protein-PINK1, which plays a pivotal role in Parkinson's disease (PD). PINK1 regulates mitochondrial stress through indirect interaction with mitochondrial proteases and the fission protein-Drp1 to further recruit Parkin by depolarized mitochondria (Chu, 2010). In addition, mitochondrial respiration complex I and IV deficiency may also contribute to the occurrence of AD and PD (Holper et al., 2019).

Comprehensive epidemiological analyses of mtDNA variations in Japanese patients with AD (*n* = 96) or PD (*n* = 96) showed that AD is uniquely associated with haplogroups G2a, B4c1, and N9b1, and PD with haplogroups M7a, M7b2, B4e, and B5b (Takasaki, 2008, 2009). In Han Chinese populations, haplogroup B5 is significantly associated with AD (*n* = 341) in patients from Southwest China (Bi et al., 2015). Cells with the B5 haplogroup had higher levels of ROS, decreased mitochondrial mass, lower ATP generation, and lower respiration when compared with non-B5 haplogroup cells (Bi et al., 2015). A study of the distribution of mtDNA haplogroups of the Han population with sporadic PD (*n* = 279) indicated that haplogroup B may confer a lower risk for PD, while haplogroup D may lead to a higher risk of PD in people younger than 50 years of age (Chen et al., 2015). Consistent with these findings, Liou et al. determined the association of mtDNA haplogroups with PD patients (*n* = 725) in Taiwan. They also found that mitochondrial haplogroup B5 confers resistance to PD. In cybrid cellular models, the B5 cybrid showed lower ROS generation and a lower rate of apoptosis compared with the B4 cybrid (Liou et al., 2016).

#### Psychiatric Disorders

Mitochondrial abnormalities may be involved in the pathophysiology of psychiatric disorders, such as schizophrenia, bipolar disorder, and attention deficit hyperactivity disorder (ADHD). Studies showed decreased protein and transcript levels of mitochondrial complex I and IV, decreased mitochondrial fusion levels, increased fission levels, and impaired OXPHOS in patients with schizophrenia or with bipolar disorder (Bubber et al., 2004; Hjelm et al., 2015; Flippo and Strack, 2017; Haghighatfard et al., 2017; Rollins et al., 2018; Holper et al., 2019).

A recent study of 11 families with schizophrenia demonstrated that mtDNA A15395G and A8536G were deleterious (Bi et al., 2016). Functional characterization further confirmed the potential pathogenicity of the two variants which includes lower mitomass, mtDNA copy number, respiration, ATP, and higher ROS (Bi et al., 2016). The T3644C mutation was found in Japanese patients with bipolar disorder (*n* = 199) but not in healthy controls. This mutation converts a well-conserved valine, to alanine in the complex I ND1 subunit, and may impair assembly of complex I. The m.3644T>C (MT-ND1) variant alters mitochondrial function by decreasing mitochondrial membrane potential (MMP) and complex I activity in 3644C cybrids compared with 3644T cybrids (Munakata et al., 2004). An epidemiologic study of Korean ADHD children (*n* = 150) revealed that haplogroup B4 increases the occurrence of ADHD, and haplogroup B5 and D4b are significantly associated with ADHD boys and girls, respectively. These results suggest that mtDNA plays an important role in the genetic etiology of ADHD in Korean children (Hwang et al., 2018). Cybrids of the SH-SY5Y neuroblastoma cell line showed decreased complex V activity and MMP, but elevated oxidative stress (Verma et al., 2016).

#### Optic Neuropathy

Mitochondrial dysfunction can also cause optic neuropathy. The relationship between several mtDNA variants (G11778A, G3460A, T14484C, G11696A, G13708A, G10680A, and T12338C) and Leber's hereditary optic neuropathy (LHON) have been reported (Yoneda et al., 1989; Hotta et al., 1995; Brown et al., 2000; Ji et al., 2008). The typical LHON-related G11778A mutation in different families belongs to the Chinese haplogroups B5b, G2a, C4a1, M7b102, and M8a; the Thai urban population haplogroups M and B; and European haplogroups J, K, and H, respectively. Several groups constructed cybrids of LHON probands carrying the G3460A, G11778A, and T14484C LHON primary mutations to confirm that mitochondrial dysfunction is caused by these mtDNA variants. They detected complex I-dependent defects in respiration, decreased ATP synthesis, increased ROS production, disrupted glutamate transport, increased mitochondrial-dependent apoptotic death, delayed complex I assembly kinetics, and instability of complexes III and IV (Ghelli et al., 2003; Baracca et al., 2005; Floreani et al., 2005; Pello et al., 2008). The mtDNA G13051A leaded to variable neurology and activated mitophagy in LHON patients (Dombi et al., 2016). Mitophagy activation can also repair LHON-associated mitochondrial dysfunction and improve cell survival (Sharma et al., 2019).

These results point to promising targets for predicting the probabilities and initial diagnosis of nervous system diseases, although the etiology of these diseases remains unclear. Targeting mitochondrial function and oxidative stress, with antioxidants, coenzyme Q10, and vitamin C may be effective strategies to ameliorate the progress of nervous system diseases.

### Endocrine System Diseases

Steroid hormone biosynthesis is carried out in mitochondria. The ATP produced by the mitochondria also provides energy for hormone generation and trafficking. mtDNA variants may result in endocrine organ defects due to impaired OXPHOS (Chow et al., 2017).

#### Diabetes Mellitus

Diabetes mellitus (DM) is a major global health problem. It is a challenge to understand the physiological and pathological conditions that lead to the development of this disease (Zimmet et al., 2001; Roglic and Unwin, 2010). Mitochondria are essential for providing energy to maintain insulin metabolism; mtDNA mutations of OXPHOS complexes can lead to pancreatic islet dysfunction. Increased mitochondrial fission may impair endothelial function via increased ROS in DM (Shenouda et al., 2011). Decreased OXPHOS and fatty acid oxidation in insulin-sensitive tissues has been reported (Kwak and Park, 2016). Mitochondrial uncoupling may protect the mitochondrial matrix against lipid-induced mitochondrial damage (Schrauwen and Hesselink, 2004).

The association between mtDNA haplogroups and the risk of DM is controversial. Some studies found diabetes susceptibility genes located in mitochondria-encoded genomes, such as G3316A and C3310T in a Japanese family (Nakano et al., 1998; Hattori et al., 2005), and G3316A, C3310T, A3243G T3394C, G4491A, T16189C, and T16519C in a Chinese population (*n* = 826) (Liao et al., 2008; Li M. Z. et al., 2008; Wang et al., 2013; Zhong et al., 2014). The N9a, M8a, B4 and D4 haplogroups appeared to be related to DM in East Asia (*n* = 1289) (Fuku et al., 2007; Loo et al., 2014; Li et al., 2015). A cybrid with a C3310T mutation showed that mitochondrial complex I activity, ATP generation, oxygen consumption were significantly decreased (Chen et al., 2006). Fuku et al. found that mitochondrial haplogroup N9a was a significant protective factor for DM in a Korean study (*n* = 732) (Fuku et al., 2007). In contrast, Niu et al. found that N9a was a risk factor for diabetic nephropathy (*n* = 235) (Niu et al., 2015). Subsequently, Fang et al. confirmed that the N9a haplogroup increased the risk of DM in the Chinese population by altering mitochondrial function and intracellular mitochondrial signals. The N9a haplogroup cybrids exhibited lower respiratory chain complex activity, ATP, MMP and oxygen consumption; however, they contained more ROS and fragmented mitochondria than non-N9a haplogroup cybrids. Insulin-stimulated glucose uptake was partially inhibited through enhanced stimulation of ERK1/2 phosphorylation and subsequent TLR4 activation in N9a haplogroup cybrids (Fang et al., 2018). Taken together, these studies show that mitochondrial haplogroups profoundly affect the occurrence and development of diabetes. Some antioxidants, including vitamin C and vitamin E, can ameliorate the oxidative stress associated with diabetes (Victor et al., 2011).

#### Diabetic Kidney Disease

Diabetic kidney disease (DKD) is the most common cause of end-stage kidney disease worldwide. Mitochondrial dysfunction plays a role in the pathophysiology of diabetes (Susztak et al., 2006; Fakhruddin et al., 2017; Forbes and Thorburn, 2018). Overproduction of ROS, activation of apoptosis, and defective mitophagy have been shown to contribute to the progression of the disease (Wei and Szeto, 2019). A study of mitochondria-targeted metabolic tubular injury in diabetic kidney disease, which included healthy controls (*n* = 65), diabetes patients without kidney disease (*n* = 48), and DKD patients (*n* = 60) was carried out in China. The accumulation of damaged mtDNA, fragmented mitochondria, activated apoptosis, loss of MMP, and perturbations in glycolysis and TCA cycle were detected in tubules and PBMCs from the patients. These results indicate that mitochondrial damage may be the hallmark of DKD patients (Jiang et al., 2019).

Salidroside, an active component from the traditional Chinese medicine Rhodiola rosea L, stimulated the Sirt1/PGC-1alpha axis and ameliorated diabetic nephropathy through enhanced mitochondrial DNA copy number and ETC protein expression in mice (Xue et al., 2019). SIRT3 overexpression also inhibited kidney tumor cells and improved mitochondrial biogenesis rather than exhibiting the Warburg effect (Liu et al., 2018). Overexpression of MnSOD can abrogate mitochondrial dysfunction and effectively prevent the development of diabetic retinopathy (Madsen-Bouterse et al., 2010). Pyruvate kinase M2 activation may protect against the progression of diabetic glomerular pathology and mitochondrial dysfunction by increasing glucose metabolic flux, and inhibiting the production of toxic glucose metabolites (Qi et al., 2017).

#### Obesity

Obesity is one of the most important pathogenic factors of DM. Impaired mitochondrial lipid oxidation, increased inflammation, and increased mitochondrial OXPHOS in the liver have been observed in obese subjects (Khasawneh et al., 2009; Rogge, 2009; Buchner et al., 2011). Guo et al. found that the C8684T transition of haplogroup M8a, and the C3497T and T1119C transitions of haplogroup B4c caused increased susceptibility to DM.

#### Asthenozoospermia

Asthenozoospermia is a multi-factor disorder that affects approximately half of males with infertility, and nearly 15% of cases result from genetic abnormalities (Moore and Reijo-Pera, 2000; Ferlin et al., 2007). Mitochondrial ATP, appropriate MMP levels, respiration activity, and low ROS levels are necessary to sustain normal sperm motility (Kasai et al., 2002; Marchetti et al., 2002; Ferramosca et al., 2012). The first study of human mtDNA haplogroups associated with asthenozoospermia was performed in Spain. The authors showed that haplogroups H and T conferred susceptibility to non-asthenozoospermic and asthenozoospermic, respectively. More importantly, complex IV activity was significantly decreased in haplogroup T cybrids (Ruiz-Pesini et al., 2000). In the Han population, men with haplogroup R exhibited decreased frequency of asthenozoospermia (*n* = 312) (Feng et al., 2013). Recently, Wang's group reported that haplogroup M8a played a critical role in the penetrance of variant C8684T in the pathogenesis of non-obstructive azoospermia due to increased mtDNA damage and impaired normal spermatogenesis (Ji et al., 2018). These studies indicated that mitochondria play a pivotal role of human fertility.

### Cancer

Cancer is a chronic non-communicable disease affecting most of the people worldwide. Cancer cells attack the immune system of the human body. Mitochondria play a critical role in providing energy for T cells, B cells, and marcrophages, albeit the metabolism of the various cells are diverse. For example, M1 macrophages utilize a modified TCA cycle to drive inflammation and are characterized by increased lactate production. However, M2 macrophages require both mitochondrial TCA metabolism and glycolysis; they use acetyl-CoA to drive forward flux through the ETC, but also require glycolysis, in part to support hexosamine biosynthesis (Vats et al., 2006; Haschemi et al., 2012; Huang et al., 2014; Jin et al., 2014). Each cell type possesses its own unique immune status, it can be pro-inflammatory or anti-inflammatory, depending on the different metabolic pathways. Additionally, new mitochondrial DNA synthesis enables NLRP3 inflammasome activation (Zhong et al., 2018). Therefore, mtDNA mutations profoundly affect the human immune system. Here, we focus on some common diseases associated with cancer.

The “Warburg effect” is a feature of most cancer cells, they prefer aerobic glycolysis from glucose to lactate in the presence of oxygen, rather than complete oxidation of glucose and mitochondrial respiration (Warburg, 1956; Wallace, 2005). Mitochondrial DNA mutation would affect mitochondrial biogenesis and turnover, mitophage, fission and fusion dynamics, oxidative stress, and cell death (Smith et al., 2012). The balance of mitochondrial metabolism plays a crucial role in the pathogenesis and progression of human malignancies, and mtDNA mutations play bidirectional roles in tumorigenesis; some are protective factors, while others are risk factors (Xu et al., 2006; Li et al., 2007; Ma L. et al., 2018).

#### Endometrial Cancer

A PGC1α-dependent pathway increases mitochondrial biogenesis, mitochondrial fission, mitophagy, proteolysis, and antioxidant response in endometrial cancer (Cormio et al., 2017), and haplogroup D shows significant correlation with the incidence of endometrial cancer in the Yunnan province in China (Xu et al., 2006). Treatment of 1, 1-bis (3′-indolyl)-1-(p-substituted phenyl) methane has been reported to decrease mitochondrial membrane potential and induce apoptosis in endometrial cancer cell lines (Hong et al., 2008).

#### Breast Cancer

Mitochondrial dysfunction and abnormal intracellular mitochondrial signaling has been detected in breast cancer cells (Santidrian et al., 2013; Pelicano et al., 2014). Haplogroup M may be a risk factor for breast cancer. Mutations in the D-loop region are more likely to be detected in benign breast tumors (*n* = 104) (Fang et al., 2010). The frequency of haplogroup D5 is significantly increased in patients with breast cancer. It was found that mitochondrial respiration, ATP content, and MMP levels were decreased in D5 haplogroup cybrids compared to those with non-D5 haplogroups. The D5 cybrids were also more susceptible to tumorigenesis through activation of the AKT pathway, mediated by ROS generation (Ma L. et al., 2018). A potential therapeutic strategy for breast cancer may depend on improving the NAD+/NADH balance through treatment with NAD+ precursors, which can inhibit metastasis and prevent progression of breast cancer (Santidrian et al., 2013).

#### Cervical Cancer

mtDNA variations located in the D-loop, coding region, and tRNA and rRNA genes are potential biomarkers in cervical carcinogenesis (Kabekkodu et al., 2014). Two groups investigated mtDNA mutations in cervical cancers of Chinese women. Zhai et al. reported that an mtDNA C150T polymorphism in HPV-positive cervical cancer patients was significantly increased compared to HPV-negative controls (Zhai et al., 2011). Li's group found that mitochondrial haplogroup D4b1 enhanced the risk of cervical cancer initiation in Chinese women (*n* = 150) (Li et al., 2016).

There are many therapeutic drugs targeted to mitochondrial dysfunction available today: benzimidazolethiol induces apoptosis by regulating the PI3K/Akt signaling pathway, interferon alpha activates both the intrinsic mitochondrial pathway and endoplasmic reticulum stress-induced pathway, mefloquine impairs mitochondrial function and inhibits mTOR pathway, nicotinamide induces mitochondrial-mediated apoptosis through oxidative stress, tocotrienol inhibits proliferation and inducing apoptosis, betulinic acid induces apoptosis by regulating PI3K/Akt signaling, and pterostilbene targets m-TOR/PI3K/Akt signaling pathway via disruption of MMP (Hoti et al., 2003; Shi et al., 2016; Feng et al., 2017; Li et al., 2017; Xu T. et al., 2017; Xu W. et al., 2017; Hong Bin et al., 2018; Tian et al., 2018).

#### Prostate Cancer

Prostate cancer is associated with dysregulation of OXPHOS. A multiethnic cohort epidemiological study of 4,086 prostate cancer cases and 3,698 controls from African, Asian, American, European, Latino, and Native Hawaiian patients was performed in order to examine the association of mtDNA and prostate cancer. This study revealed that haplogroup N contributed to overall prostate cancer, however, the mtDNA-encoded OXPHOS genes were not associated with prostate cancer risk in this cohort (Giorgi et al., 2016). A study of a Korean population also revealed no association of the mtDNA-encoded OXPHOS genes with prostate cancer (*n* = 139) (Kim et al., 2008). However, several studies of prostate cancer demonstrated that mitochondrial dysfunction and altered intermediary metabolism, especially high ROS levels, occurred in prostate cancer cells (Dakubo et al., 2006; Mizumachi et al., 2008; Altieri, 2010; Chaudhary et al., 2017). ROS production accelerated mtDNA mutations in prostate cancer and further stimulated malignant transformation of prostate through increased ETC activity (Dakubo et al., 2006).

#### Lung Cancer

Cell migration and invasion, which occurs through the induction of AKT and AMPK pathways in lung cancer cells, has been associated with mitochondrial dysfunction (Han et al., 2018). Two case-control cohort studies found an association between mtDNA variation and lung cancer risk in a Han Chinese population from southwestern China (*n* = 422). Zheng et al. revealed that haplogroups D and F were protective factors for lung cancer, while haplogroups G and M7 increased susceptibility (Zheng et al., 2012). However, Fang's group demonstrated that haplogroups F and G predisposed people to lung cancer (*n* = 237). Although the results varied, both studies suggest that haplogroup G is a risk factor for lung cancer due to excess ROS generated by the impaired mitochondrial respiration chain (Fang Y. et al., 2015). In lung cancer, altered rates of mitochondrial fission and fusion were seen, which can influence metabolic function, proliferation, and cell survival. Therefore, altering mitochondrial dynamics may be a therapeutic strategy, for example, inhibiting mitochondrial fission can prevent cell cycle progression in lung cancer (Rehman et al., 2012; Lennon and Salgia, 2014).

#### Hepatocellular Carcinoma

Mutations in the mitochondrial D-loop region have been reported in hepatocellular carcinoma (HCC), which may partly contribute to cancer development (Zhang et al., 2010). Mitochondrial pyruvate carrier (MPC1/2) protein expression was significantly downregulated in HCC, and may serve as a biomarker for the identification of patients with this disease (Ma X. et al., 2018). Mitochondrial fission significantly promoted the reprogramming of focal-adhesion dynamics and lamellipodia formation in HCC cells, mainly by activating Ca^2+^/CaMKII/ERK/FAK pathway (Sun et al., 2018). In addition, Drp1-mediated mitochondrial fission promoted cell proliferation through crosstalk between the p53 and NF-kappaB pathways in HCC (Zhan et al., 2016). Therefore, treatment with mitochondrial division inhibitor-1 may decrease proliferation in HCC cells.

#### Esophageal Cancer

Esophageal cancer (EC) has a very high mortality rate in China. Casticin treatment plays a pivotal role in inhibiting proliferation and inducing apoptosis of EC cells through activation of JNK signaling pathway, and hesperetin induces apoptosis via increased intracellular reactive oxygen species (Wu et al., 2016; Qiao et al., 2019). A study of mitochondrial haplogroups and esophageal cancer (*n* = 30) in the Taihang Mountain and Chaoshan areas of China has shown that haplogroups D4a and D5 in Taihang Mountain, and haplogroups D and D5 in Chaoshan areas, were related to higher susceptibility to esophageal cancer (Li et al., 2007). Overall, haplogroup D, specifically sub-haplogroups D4a and D5a, can serve as potential biomarkers for esophageal cancer, at least in these two areas. Other papers showed that haplogroup D4a was associated with an increased risk of thyroid cancer (*n* = 100) in China (Fang et al., 2010). As previously mentioned, haplogroup D5 was also a risk factor of breast cancer. It appears that haplogroup D is a risk factor for a diverse group of diseases because it impairs mitochondrial OXPHOS.

### Cardiovascular and Cerebrovascular Diseases

Cardiovascular and cerebrovascular diseases are major health problems worldwide, but Asian countries have higher mortality rates for stroke than Western countries, although these rates have recently decreased in Japan and urban areas in China. South Asian, but not East Asian countries have a higher mortality rate for coronary heart disease than Western countries (Zhang et al., 2007; Ueshima et al., 2008). There are about 290 million patients, or one in five adults, with cardiovascular or cerebrovascular diseases in China (Wallace, 2000).

#### Hypertension

Hypertension is one of the most common risk factors of cardiovascular disease, affecting ~168.1 million in China (Wang et al., 2019). It can be caused by both hereditary and genetic factors. Mutations in the mitochondrial genome are associated with essential hypertension; several mtDNA mutations associated with hypertension are found in haplogroup D4j (A4295G) (Li Z. et al., 2008), haplogroup G2a1 (A4435G) (Lu et al., 2011), and haplogroup B5b1 (T16189C) (Zhu et al., 2016). Mitochondrial dysfunction associated with increased ROS production may be involved in the pathogenesis of hypertension (Dikalov and Ungvari, 2013; Lahera et al., 2017). Lymphocyte cell lines with a tRNA(Met) C4467A mutation showed oxidative stress and mitochondrial biogenesis dysfunction, including lower ATP generation, MMP activity, and oxygen consumption, and increased ROS levels (Liu et al., 2017).

#### Myocardial and Cerebral Infarction

Myocardial and cerebral infarction are multifactorial disorders affected by both genetic and environmental conditions, such as coronary atherosclerosis (Sawabe et al., 2011), carotid artery stenosis (Iizuka et al., 2009), hypertrophic cardiomyopathy (Wei et al., 2009), and left ventricular non-compaction (Tang et al., 2010). Mitochondria-derived ROS plays a role in myocardial and cerebral infarction due to the fact that mitochondria in vascular endothelial cells are the major source of superoxide (Guzik et al., 2006). Therefore, it is important to determine the variants of mtDNA associated with myocardial and cerebral infarction. In the Japanese population, mtDNA C5178A transversion causes leucine to methionine substitution in ND2, resulting in anti-atherosclerotic effects in diabetic subjects and a lower prevalence of myocardial infarction (Takagi et al., 2004). The study found that mitochondrial haplogroup A contributes to atherothrombotic cerebral infarction (*n* = 1,181), but only in females. Haplogroup N9b protects against myocardial infarction; however, haplogroup G1 is a risk factor for this disease in Japanese males (Nishigaki et al., 2007a,b). Mitochondrial haplogroups A and M7a increase the risk for coronary atherosclerosis in a Japanese population (*n* = 1,536). Surprisingly, a haplogroup associated with extreme longevity, D4a, conferred a risk of myocardial infarction (Alexe et al., 2007; Cai et al., 2009; Sawabe et al., 2011). Inhibition of mitochondrial permeability transition improved functional recovery and reduced mortality following acute myocardial infarction in mice (Gomez et al., 2007). Therefore, targeting mitochondrial calcium transport and inhibiting mitochondrial fission may be effective strategies for myocardial infarction (Cooper and Eguchi, 2018; Frangogiannis, 2018).

#### Hypertrophic Cardiomyopathy

Hypertrophic cardiomyopathy (HCM) is a common genetic disorder, affecting 1 in 500 individuals worldwide (Maron et al., 2006). Mitochondrial haplogroup M10 may be a risk factor for HCM, specifically, three mutations - G7967A in the COX II of complex IV, and T12477C and G13135A in the ND5 of complex I. Mitochondrial complex I activity was markedly decreased in the HCM individuals, resulting in disrupted mitochondrial respiratory function (Wei et al., 2009). The mitochondrial ND5 T12338C variant which belongs to haplogroup F2a was associated with hypertrophic cardiomyopathy in a Chinese pedigree (Liu et al., 2012). A characteristic T2336C homoplasmic mutation in the mitochondrial 16S rRNA gene of HCM has also been found in a Chinese family. Reduced ATP and MMP levels, and increased ROS generation in the mutant 2336C cybrids may lead to deterioration of mitochondrial function (Li et al., 2018).

#### Left Ventricular Non-compaction

Left ventricular non-compaction (LVNC) is a genetically heterogeneous pathological disorder that is affected by both the nuclear and mitochondrial genomes, leading to congenital heart disease (Digilio et al., 1999; Xing et al., 2006). Tang et al. found that mtDNA A3397G and T3398C of the complex I ND1 subunit may disrupt mitochondrial function to initiate LVNC (Tang et al., 2010).

#### Stroke

Stroke is a complex multifactorial disorder caused by both genetics and environment in the Chinese population (Della-Morte et al., 2012). One study shows that mtDNA C5178A belongs to haplogroup D4b and is a protective factor for IS in the Chinese Han population (*n* = 200) (Yang et al., 2014). Other studies reported that m.5178C, but not m.5178A, is a risk factor for several diseases including myocardial infarction (*n* = 517), cerebrovascular diseases (*n* = 127), and diabetes (*n* = 270) (Wang et al., 2001; Ohkubo et al., 2002; Takagi et al., 2004). One explanation may be that the production of ROS in complex I disrupts the structure and respiratory chain function, and further damages the cardiovascular system. Rapamycin treatment attenuated mitochondrial dysfunction following cerebral ischemia, possibly through enhancement of mitophagy (Li et al., 2014).

## Conclusions

In summary, the studies described in this review shed light on the pathogenesis of diseases associated with mitochondrial dysfunction. They provide biological plausibility for the observed epidemiological surveys, although a number of limitations must also be considered; notably, the specific mechanisms of disease require further investigation. An intriguing finding is that the same mitochondrial haplogroup can have negative or positive association with different diseases. These associations remain unclear, although haplogroups defined by using common SNPs in each ethnic group may be a potential explanation. The distribution of mtDNA allele frequency varies considerably with the populations studied, which implies that haplogroup analysis is insufficient across populations. The results suggest that there are mechanisms other than mitochondrial pathways that affect susceptibility to these diseases. Mitochondrial retrograde signaling pathways may have profound effects on nuclear-mitochondrial interactions in cellular morphology and functionality. A possible explanation is that the nucleus attempts to make more mitochondria compensate for the energy deficiency. Other complex networks including proteostasis, mitochondrial unfolded protein response (UPR) and ROS signaling may also play pivotal roles in the organism's metabolism and deserve future investigation.

## Author Contributions

DS and H-XZ designed this review. DS searched literature and wrote the initial manuscript. YW made the table. JW and LJ supervised and provided critical comments on the manuscript. DS, YW, H-XZ, LJ, and JW read, amended, and discussed the article.

### Conflict of Interest

The authors declare that the research was conducted in the absence of any commercial or financial relationships that could be construed as a potential conflict of interest.
